# Postmortem investigation of fatalities following vaccination with COVID-19 vaccines

**DOI:** 10.1007/s00414-021-02706-9

**Published:** 2021-09-30

**Authors:** Julia Schneider, Lukas Sottmann, Andreas Greinacher, Maximilian Hagen, Hans-Udo Kasper, Cornelius Kuhnen, Stefanie Schlepper, Sven Schmidt, Ronald Schulz, Thomas Thiele, Christian Thomas, Andreas Schmeling

**Affiliations:** 1 Institute of Legal Medicine, University Hospital Münster, Münster, Germany; 2 Institute of Immunology and Transfusion Medicine, University Medicine of Greifswald, Greifswald, Germany; 3 Institute of Pathology at Clemens Hospital Münster, Münster, Germany; 4 Institute of Neuropathology, University Hospital Münster, Münster, Germany

**Keywords:** SARS-CoV-2, COVID-19, Vaccination, Fatalities, Autopsy, Myocardits, VITT

## Abstract

Thorough postmortem investigations of fatalities following vaccination with coronavirus disease 2019 (COVID-19) vaccines are of great social significance. From 11.03.2021 to 09.06.2021, postmortem investigations of 18 deceased persons who recently received a vaccination against COVID-19 were performed. Vaxzevria was vaccinated in nine, Comirnaty in five, Spikevax in three, and Janssen in one person. In all cases, full autopsies, histopathological examinations, and virological analyses for the severe acute respiratory syndrome coronavirus 2 were carried out. Depending on the case, additional laboratory tests (anaphylaxis diagnostics, VITT [vaccine-induced immune thrombotic thrombocytopenia] diagnostics, glucose metabolism diagnostics) and neuropathological examinations were conducted. In 13 deceased, the cause of death was attributed to preexisting diseases while postmortem investigations did not indicate a causal relationship to the vaccination. In one case after vaccination with Comirnaty, myocarditis was found to be the cause of death. A causal relationship to vaccination was considered possible, but could not be proven beyond doubt. VITT was found in three deceased persons following vaccination with Vaxzevria and one deceased following vaccination with Janssen. Of those four cases with VITT, only one was diagnosed before death. The synopsis of the anamnestic data, the autopsy results, laboratory diagnostic examinations, and histopathological and neuropathological examinations revealed that VITT was the very likely cause of death in only two of the four cases. In the other two cases, no neuropathological correlate of VITT explaining death was found, while possible causes of death emerged that were not necessarily attributable to VITT. The results of our study demonstrate the necessity of postmortem investigations on all fatalities following vaccination with COVID-19 vaccines. In order to identify a possible causal relationship between vaccination and death, in most cases an autopsy and histopathological examinations have to be combined with additional investigations, such as laboratory tests and neuropathological examinations.

## Introduction

Vaccination with potent and well-tolerated vaccines against coronavirus disease 2019 (COVID-19) is considered an effective measure to contain the coronavirus pandemic. At the same time, the vaccines are intended to prevent severe acute respiratory syndrome coronavirus 2 (SARS-CoV-2) infections, or at least severe or fatal COVID-19 infections. So far, four COVID-19 vaccines have been approved by the European Medical Agency (EMA), all encoding the spike protein antigen of SARS-CoV-2. Those are two mRNA-based vaccines: BNT162b2 (Comirnaty, BioNTech/Pfizer) and mRNA1273 (Spikevax, Moderna); and two recombinant vector-based vaccines: adenovirus type 26 vector COVID-19 Vaccine Janssen (Janssen, Johnson&Johnson), and the recombinant chimpanzee adenoviral vector vaccine ChAdOx1 nCoV-19 (Vaxzevria, AstraZeneca). As of June 30, 2021, a total of 74,871,502 vaccinations have been administered in Germany, including 54,898,640 with Comirnaty, 11,570,155 with Vaxzevria, 6,471,052 with Spikevax, and 1,931,655 with Janssen. A total of 30,986,128 people were fully vaccinated [48]. Due to the inherently limited number of people that were studied in the initial trials, it is necessary to further monitor adverse events after vaccination during population wide roll out of COVID-19 vaccines to detect also very rare adverse events. This allows ongoing benefit-risk assessments of the approved vaccines.

Since end of February 2021, venous thromboses at unusual sites including cerebral venous thrombosis (CVT) and/or splanchnic vein thrombosis in combination with moderate to severe thrombocytopenia occurred in several hundred individuals typically 4 to 30 days after vaccination with Vaxzevria and Janssen [27, 39, 55–57]. Immunoglobulin G class platelet-activating antibodies directed against platelet factor 4 (PF4; CXCL4) were identified as underlying cause, reflecting a close relationship to autoimmune Heparin-induced thrombocytopenia (HIT) [27]. The underlying syndrome has been designated vaccine-induced immune thrombotic thrombocytopenia (VITT); other names are thrombosis with thrombocytopenia syndrome (TTS), a term favored by some reporting agencies; vaccine-associated (immune) thrombotic thrombocytopenia (VATT); and the original term vaccine-induced prothrombotic immune thrombocytopenia (VIPIT) [24].

Besides VITT, other immune syndromes were reported following COVID-19 vaccination including thrombotic thrombocytopenic purpura/hemolytic uremic syndrome, (auto-)immune thrombocytopenia or Guillain-Barré Syndrome. Furthermore, peri-/myocarditis was reported as a rare event following vaccination with mRNA-based vaccines [48].

In Germany, the Paul Ehrlich Institute is responsible for the registration of adverse events after vaccination. The Paul Ehrlich Institute summarizes all reports it receives, irrespective of a causal relationship with vaccination, and regularly issues safety reports [48]. The Paul Ehrlich Institute received reports of 873 deaths with a temporal relation to COVID-19 vaccination by May 31, 2021. Among the 873 reported deaths, 73 were related to the COVID-19 disease and not to adverse events. The vast majority of those who died had multiple comorbidities, such as carcinomas, renal failure, heart diseases, and atherosclerotic changes, which are potential causes of death. A total of 21 patients vaccinated with Vaxzevria died as a result of VITT.

A total of 25 fatal hemorrhages, 24 of which were cerebral, were reported after vaccination with Vaxzevria. A total of 27 fatal hemorrhages occurred after Comirnaty vaccination, 18 of which were cerebral hemorrhages. A total of 2 fatal hemorrhages were reported after Spikevax vaccination.

There were 5 deaths reported with myocarditis ranging from 1 to 50 days after vaccination with Corminaty. All 5 individuals had preexisting cardiovascular diseases that were potential causes of death. There was one patient who died 2 days after Vaxzevria vaccination from septic shock, acute renal failure, and myocarditis. Anaphylactic reactions were reported in 293 cases. Among these, 94 cases had the highest level of diagnostic safety. No fatal anaphylaxis was reported [47].

In cases of deaths with a temporal relation to vaccinations, autopsies together with additional investigations can provide an important contribution for clarifying the causal relationship between vaccination and death. While numerous studies have been published on postmortem investigation of COVID-19 [3, 8, 19–21, 25, 32, 38, 60, 62], there are only a few publications on postmortem diagnostics of adverse events after vaccination [22, 29, 50]. In addition to clinical pathologists, forensic pathologists are also tasked with postmortem investigations of fatalities following vaccination, making the topic of this article relevant to legal medicine. In the following, we report on the postmortem investigations of 18 fatalities after vaccination with COVID-19 vaccines. The possibilities and limitations of postmortem diagnostics of fatal adverse events after vaccination are discussed.

## Material and methods

From 11.03.2021 to 09.06.2021, postmortem investigations of 18 persons who recently received a vaccination against COVID-19 were performed on behalf of the public prosecutor's offices in Bielefeld, Detmold, and Münster. The catchment area of these public prosecutor’s offices comprises around 3.24 million people, which accounts for about 4% of Germany’s population [58, 59].

The following data were available for evaluation: case histories provided by the investigating authorities, medical records for in-hospital deaths, autopsy protocols, and reports on additional investigations (laboratory tests, histology, neuropathology).

The autopsies were performed in accordance with the guidelines of the German Society of Legal Medicine for forensic autopsies [7]. In addition to the usual samples for histological examinations, pharyngeal mucosa, the injection site, as well as lymph nodes of the axillary region, and a sample of the deltoid muscle of the site of vaccination were taken. Swabs of the naso-pharynx and bronchial mucosa were obtained for virological analysis for SARS-CoV-2. In addition to the usual samples for forensic toxicological and metabolic analyses, serum extracted from femoral vein blood was obtained to be analyzed for anaphylaxis and VITT. In order to assess anaphylaxis, serum tryptase, total IgE, interleukin 6, CRP, complement factor 3, and complement factor 5 were quantified. In cases of suspected VITT, anti-PF4 antibody tests and platelet activation tests were performed in the platelet laboratory in Greifswald as described by Handtke et al. [28].

The following data were gathered in a registry: age and gender of the deceased, vaccine, 1st or 2nd vaccination, time interval between vaccination and death, place of death, findings of the autopsy and the additional investigations, cause of death, and the assessment of the possible causal relationship between vaccination and death.

## Results

Within the study period, postmortem investigations were performed in 18 fatalities (9 females) in temporal relationship to COVID-19 vaccination. Table 1 provides detailed information concerning all cases.

**Table 1:** Detailed information concerning the investigated 18 fatalities

Case nr	Gender	Age	Vaccine	1 or 2 vaccinations	Time interval between vaccination and death (in days)	Place of death	Relevant findings of postmortem investigations	Cause of death	Assessment of causal relationship between vaccination and death
1	m	82	Spikevax	1	1	Home	Severe coronary sclerosis, massive cardiac hypertrophy, extensive myocardial infarction scars, anaphylaxis diagnostics negative	Most likely severe pre-existing cardiac changes with infarction scars	No evidence
2	f	91	Spikevax	1	1	Home	Severe coronary sclerosis, massive cardiac hypertrophy, myocardial infarction scars, anaphylaxis diagnostics negative	Most likely severe pre-existing cardiac changes with infarction scars	No evidence
3	f	32	Vaxzevria	1	12	Home	Massive cerebral hemorrhage, anti-PF4 heparin antibody tests: positive, HIPA-Test: positive, PIPA-Test: positive	Massive cerebral hemorrhage	Very likely
4	f	34	Vaxzevria	1	1	Home	Obesity, massive cardiac hypertrophy, myocardial infarction scars, fresh myocardial infarction, anaphylaxis diagnostics negative	Recurrent myocardial infarction in the presence of massive cardiac hypertrophy	No evidence
5	f	48	Vaxzevria	1	10	Workplace	Aortic dissection with rupture, high blood loss	Bleeding from ruptured aorta	No evidence
6	m	65	Comirnaty	1	1	Home	Severe coronary sclerosis, massive cardiac hypertrophy, myocardial infarction scars, myocarditis, anaphylaxis diagnostics negative	Myocarditis in the presence of severe pre-existing cardiac changes	Possible
7	m	71	Comirnaty	1	1	Home	Massive cardiac hypertrophy, coronary sclerosis, anaphylaxis diagnostics negative	Most likely severe pre-existing cardiac changes with infarction scars	No evidence
8	f	57	Spikevax	2	6	Home	Severe coronary sclerosis, fatty liver, high levels of glucose and lactat in CSF and aqueous humor exceeding the cumulative levels of Traub	Hyperglycemic coma	No evidence
9	m	63	Vaxzevria	1	14	Home	Severe coronary sclerosis, cardiac hypertrophy, myocardial infarction scars, liver cirrhosis	Most likely severe pre-existing cardiac changes	No evidence
10	m	61	Vaxzevria	1	1	Home	Severe coronary sclerosis, massive cardiac hypertrophy, anaphylaxis diagnostics negative	Most likely severe pre-existing cardiac changes with infarction scars	No evidence
11	m	71	Vaxzevria	unknown	10	Hospital	DVT, pulmonary embolism, severe coronary sclerosis, massive cardiac hypertrophy, myocardial infarction scars, VITT-diagnostics negative	Pulmonary embolism in the presence of DVT	No evidence
12	f	38	Vaxzevria	2	8	Hospital	Multiple fresh thrombi, including in the cerebral venous sinuses, cardiac hypertrophy, fresh myocardial infarction, hypoxic brain damage, anti-PF4 heparin antibody tests: positive, HIPA-Test: positive, PIPA-Test: positive	Hypoxic brain damage following an anaphylactic reaction to anesthetics	Unlikely
13	f	72	Comirnaty	1	12	Home	Massive cerebral hemorrhage, coronary sclerosis, cardiac hypertrophy, VITT diagnostics negative	Massive cerebral hemorrhage	No evidence
14	f	65	Vaxzevria	1	10	Hospital	Signs of a bleeding diathesis, cerebral hemorrhages, CVT, mild coronary sclerosis, anti-PF4 heparin antibody tests: positive, HIPA-Test: positive, PIPA-Test: positive	CVT and cerebral hemorrhage with hypoxic brain damage	Very likely
15	m	79	Comirnaty	2	6	Home	DVT, massive pulmonary embolism, coronary sclerosis, pericarditis, chronic pulmonary emphysema, VITT diagnostics negative	Pulmonary embolism in the presence of DVT	No evidence
16	m	57	Vaxzevria	unknown	2	Hospital	Severe coronary sclerosis, massive cardiac hypertrophy, extensive myocardial infarction scars, fresh myocardial infarction	Recurrent myocardial infarction	No evidence
17	f	72	Comirnaty	2	0	Vaccination center	Severe coronary sclerosis with coronary thrombosis, myocardial infarction scars, fresh myocardial infarction, anaphylaxis diagnostics negative	Coronary thrombosis with fresh myocardial infarction	No evidence
18	m	69	Janssen	1	9	Home	CVT, severe coronary sclerosis with coronary thrombosis, massive cardiac hypertrophy, fresh myocardial infarction, anti-PF4 heparin antibody tests: positive, HIPA-Test: positive, PIPA-Test: positive	Coronary thrombosis with fresh myocardial infarction	Possible

The mean age of the deceased was 62.6 years (age range: 32–91 years). Vaxzevria was vaccinated in nine, Comirnaty in five, Spikevax in three, and Janssen in one person. The time interval between the last vaccination and death ranged from a few minutes (case 17) up to 14 days (case 9). Case 17 involved a person who collapsed in a vaccination center immediately after vaccination; prompt resuscitation efforts were unsuccessful. A total of 12 deaths occurred at home, 4 deaths at a hospital, 1 death at a vaccination center, and 1 death at work.

**Fig. 1:**
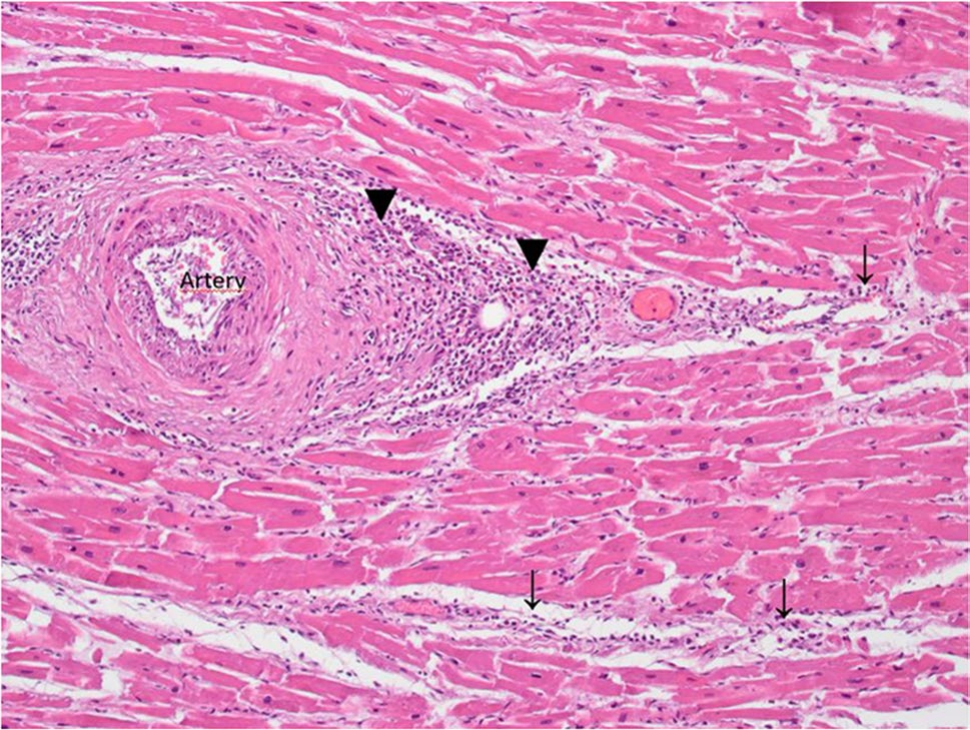
Case 6 with myocarditis with lymphocytic and plasmocytic infiltration of the perivascular space (black down-pointing triangle) and the myocard (downward arrow) (H&E, Orig. Magn. 100 ×)

In 13 deceased, the cause of death was attributed to preexisting diseases while postmortem investigations did not indicate a causal relationship to the vaccination. In one case (case 6), myocarditis was found to be the cause of death (Fig. 1). A causal relationship to vaccination is possible, but cannot be proven beyond doubt. A competing cause of death was found to be severe pre-existing cardiac changes.

**Fig. 2:**
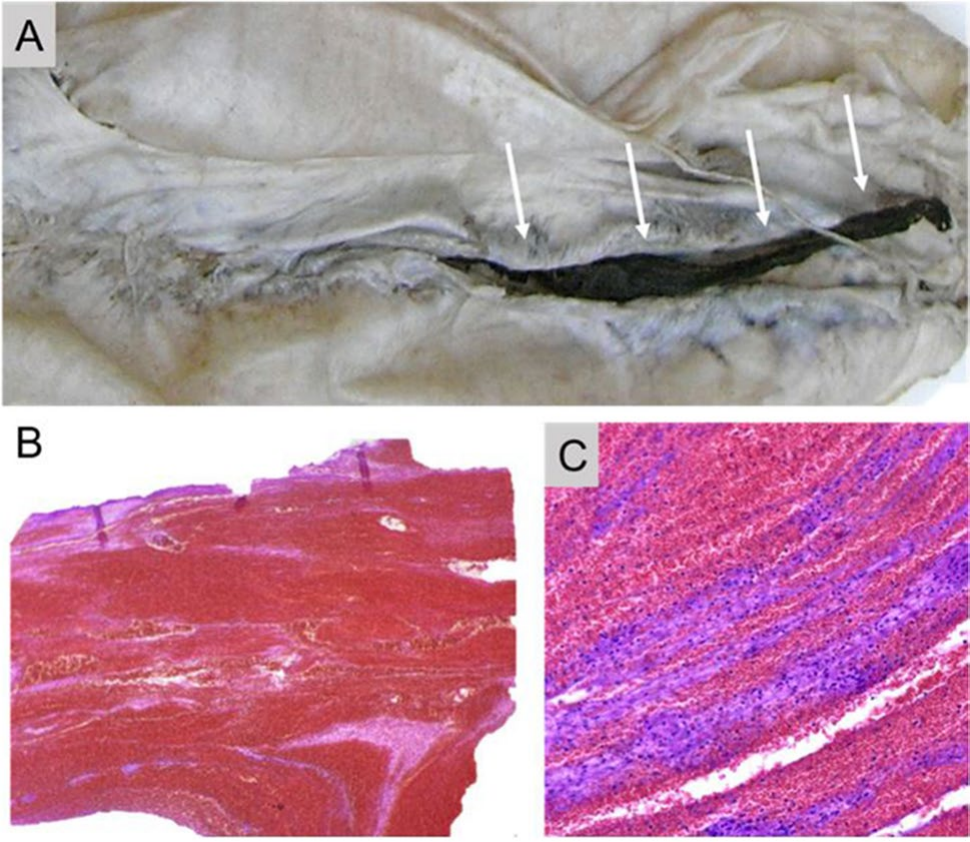
**A** The superior sagittal sinus has been partially incised showing acute, venous thrombosis (arrows). H&E sections show centrally located red blood cells/hemorrhagic areas and surrounding fibrin accumulations (**B**, Orig. Magn. 10 ×) as well as small areas with alternating lines of erythrocytes and fibrin (**C**, Orig. Magn. 40 ×)

In four cases, there was evidence of VITT (cases 3, 12, 14, and 18). In case 14, the cause of death was established in hospital, being CVT and cerebral hemorrhage with hypoxic brain damage. The diagnosis was confirmed by postmortem investigations. A second patient who died in a hospital (case 12) suffered a severe anaphylactic shock during narcosis induction, resulting in hypoxic brain damage. This patient was vaccinated 12 days before she developed the shock symptomatic. She received unfractionated heparin after the adverse event. The serum-activated platelets were stronger in presence of PF4 than in presence of PF4/heparin complexes. This suggests that the patient had true VITT antibodies related to vaccination, rather than HIT antibodies related to later heparin therapy. The autopsy revealed multiple thrombi, including in the cerebral venous sinuses. Neuropathological examination found these to be fresh thrombi that must have formed after the onset of hypoxic brain damage (Fig. 2). As thrombosis or bleeding was not involved in this death, we consider the causal relationship between vaccination and death to be unlikely. Case 3 involved a 32-year-old woman who died at home; the autopsy revealed a cerebral mass hemorrhage without CVT. Laboratory tests for VITT were positive, so a causal relationship with vaccination and death is very likely. In case 18, a 69-year-old man who also died at home, the autopsy revealed CVT and the laboratory tests for VITT were positive. However, neuropathologic changes appropriate for causing death were not detected. Furthermore, a fresh thrombus of a coronary artery with severe atherosclerosis lesions and a fresh myocardial infarction was suitable to be the cause of death. Thus, a causal relationship between vaccination and death is possible, but cannot be proven beyond doubt. Of the four cases where postmortem investigations revealed a VITT, only one had been diagnosed before death.

## Discussion

Thorough postmortem investigations of fatalities with a temporal relation to COVID-19 vaccination are of great social significance in several respects. Thus, the demonstration or exclusion of a causal relationship between vaccination and death has important implications for the benefit-risk assessment of COVID-19 vaccines and thereby serves to protect the public from unacceptable adverse effects. Furthermore, clarification of a causal relationship between vaccination and death may also be relevant for the relatives of the deceased. For example, the mother of a 32-year-old stated in a newspaper interview that received considerable media attention in Germany, that she was attacked in social media after she expressed suspicion that her daughter’s death may have been caused by vaccination. The mother was accused of lying. Some users stated that it was irresponsible to blame the death on the vaccination. The mother reported that after postmortem investigations revealed that her daughter had died of a cerebral mass hemorrhage due to VITT, she was relieved to finally have clarity [11]. Confirmation of a causal relationship between vaccination and death is also required for the enforcement of pension claims of beneficiaries. Last not least, the success of a vaccination campaign can be jeopardized by raising public concerns as to whether potential vaccine complications are thoroughly investigated or whether the results of investigations are being presented to the public in a transparent manner.

Postmortem investigations of fatalities after COVID-19 vaccination are particularly relevant with regard to the detection of anaphylaxis, VITT, and myocarditis.

### Anaphylaxis

Allergic reactions are known adverse events following immunization for virtually all vaccines, similar to other medications. These range from mild self-limited localized reactions to rare, but severe allergic reactions, including anaphylaxis. Anaphylaxis is defined as a serious systemic hypersensitivity reaction that is usually rapid in onset and may cause death [13]. The reported rate of anaphylaxis after all vaccines is estimated to be in the range of 1–10 per million doses [23, 53].

All ingredients of a vaccine may cause allergic reactions. In fact, most reactions are not caused by the active vaccine itself, but by excipients, which are inert substances added to vaccines for improving certain properties such as solubility or stability [34]. Current reviews name two excipients in COVID-19 vaccines mainly suspected for allergic reactions: polyethylene glycol in the mRNA-based vaccines and polysorbate 80 in the vector-based ones [31, 34].

The most important, albeit not the only, mechanism leading to anaphylaxis is the activation of mast cells by immunoglobulin E (IgE) antibodies against a specific antigen, which occurs within minutes or up to 4 h after exposure to the vaccine [34]. The median interval of symptom onset in an early assessment of cases for Corminaty was 13 min (range = 2–150 min), with 71% within the recommended general 15-min observation period after vaccination and 86% within 30 min [14]), which is the recommended extended observation period for individuals at greater risk for allergic reactions. However, the rare possibility of biphasic anaphylaxis has been described [16], also in the case of vaccination with Corminaty [1].

The scientific literature on vaccine-related deaths is vast. However, a majority of this literature is based on data from passive voluntary reporting systems. In contrast, the forensic literature on fatal anaphylaxis following vaccination is surprisingly scarce. In a recent review, Palmiere et al. [46] found six papers reporting cases of presumptively vaccine-related fatal anaphylaxis. Due to the limitations of five of these six papers, i.e., immunohistochemical, toxicological, or biochemical examinations being either not performed or not reported, they concluded that the diagnosis of vaccine-related fatal anaphylaxis remained hypothetical. Only in one case, comprehensive examinations, including mast cell tryptase measurement, have been performed. Death of the 3-month-old female occurred within 24 h of vaccination with hexavalent vaccine [18].

For forensic pathologists, diagnosis of anaphylaxis is challenging. Postmortem investigations should include full autopsy, histology, immunohistochemistry, and postmortem biochemistry. Macroscopical findings may include cutaneous swelling, upper airway edema, and hyperinflation of the lungs with mucus plugging [12], but are often absent [51]. Histological examinations include the pharyngeal mucosa, the spleen, the injection site, the axillary lymph nodes, and deltoid muscle of the site of injection [22]. Histology may show edema of the upper airways with eosinophilic infiltration, as well as eosinophilic accumulation and shock signs of organs in general [12, 22]. Histological and immunohistochemical examinations of splenic tissue may reveal eosinophil and activated mast cell accumulation in splenic red pulp [46]. For mast cell detection, Giemsa staining is used as well as immunostaining with anti-CD117, anti-tryptase, or anti-chymase antibodies [15, 22]. In the myocardium and coronary arteries, adventitial eosinophils and mast cells may be a potential indication of allergic coronary artery spasm [33, 46]. Postmortem biochemistry is recommended from peripheral blood, with sampling as soon as possible by aspiration from a clamped femoral or external iliac vein [26]. Increased postmortem serum mast cell tryptase levels are considered a crucial clue for the diagnosis of anaphylaxis. Total and specific IgE are indicative of an atopic disposition of the individual. Further, determination of markers of inflammation, interleukin-6 (IL-6) and C-reactive protein (CRP), and of markers of immune system activation, complement C3/C5 activity, is suggested [22].

To our knowledge, no anaphylaxis-related deaths have been reported following COVID-19 vaccination. In all deceased persons investigated in the present study who died within 1 day of vaccination, there was no evidence of an anaphylactic event. In particular in case 17, in which an anaphylactic shock was initially suspected due to the short time interval between vaccination and death, an anaphylactic reaction could be largely excluded by the autopsy as well as postmortem biochemistry and histopathological examinations.

### Vaccine-induced immune thrombotic thrombocytopenia (VITT)

VITT is characterized by thrombocytopenia, combined with thrombosis in most cases. Thrombosis can occur in both arterial and, more common, venous system. However, a distinctive feature of VITT is thrombosis in unusual locations. These include CVT, as well as splanchnic venous thrombosis [27, 55, 56].

Cases have been reported to be typically identified between 4 and 30 days after vaccination with either of two currently EMA-approved vector vaccines Vaxzevria and Janssen [39]. One single case report of a possible VITT following an mRNA-based vaccine, Spikevax, has been published to date [54]. Lack of definitive evidence in this case has been highlighted by Pishko et al. [49]. In the present study, we have found VITT in 3 deceased persons following vaccination with Vaxzevria (cases 3, 12, and 14) and 1 deceased following vaccination with Janssen (case 18). In contrast, no VITT was found following vaccination with mRNA-based vaccines in our study.

In initial reporting, female sex and younger age were proposed possible risk factors, but this may simply have reflected the demographics of the first populations to be vaccinated, i.e., often young female health care workers [27, 48, 55]. Indeed, cases in the older populations are now emerging [10] and the Paul Ehrlich Institute states a current trend into the direction of equal sex proportions [48]. We report the cases of 3 female deceased (ages 32, 38, and 65) and 1 male (age 69) with positive VITT-testing. However, it has to be emphasized that it is not possible to draw epidemiological conclusions from these data.

Death with VITT appears to occur very rarely. Up to 31.05.2021, 21 patients had been reported to the Paul Ehrlich Institute who had died of VITT in temporal relationship with a Vaxzevria vaccination [47]. In our study, there were indications of VITT in four deceased persons. It is remarkable that our study material showed an above-average number of deceased with VITT in relation to the number of inhabitants in our catchment area. If one also takes into account that presumably not all those who died after COVID-19 vaccination were autopsied in our catchment area, it is reasonable to assume that the number of deceased with VITT is significantly higher than the number of cases reported to the Paul Ehrlich Institute.

In the clinical setting, the diagnosis of VITT is made by detecting high titers of anti-PF4/heparin antibodies with an antigen-binding assay (ELISA) for PF4/heparin antibodies in combination with positive functional platelet activation assays such as the PF4 induced platelet activation test (PIPA) and the heparin induced platelet activation test (HIPA). The detection of thrombocytopenia is done by determining the platelet count in the blood [27, 44].

Postmortem, a valid determination of the platelet count is not possible if a VITT is suspected, since numerous processes take place in the blood compared to the living, which have a falsifying effect on the number of countable platelets [61]. However, PF4/heparin antibodies can be detected also in serum obtained from cadaveric blood. This examination is even possible in hemolytically altered serum. In the case of not-hemolytic serum, however, the necessary functional platelet activation assays can be performed as well. At autopsy, the cerebral sinus veins and mesenteric veins should be opened to ensure that thrombosis in these locations is not missed.

Our study shows that an autopsy and platelet laboratory testings are not sufficient to clarify a possible causal relationship between VITT and death. Indeed, it could only be decided in the synopsis of the anamnestic data, the autopsy results, laboratory diagnostic examinations, and histopathological and neuropathological examinations that VITT was very probably the cause of death in only two of our four cases. In the other two cases, no neuropathological correlate of VITT explaining death was found, while possible causes of death emerged that were not necessarily attributable to VITT.

### Myocarditis

The first reports concerning cases of myocarditis temporally linked to the vaccination with the mRNA vaccines from Comirnaty and Spikevax came from the Israeli Ministry of Health and the U.S. Department of Defense [4]. To date, there are single case reports and case series published describing myocarditis following vaccination with Comirnaty or Spikevax with rather mild courses [2, 4, 9, 17, 30, 37, 40–43, 52].

Viruses are commonly the cause of myocarditis. In addition to other infectious agents, however, drugs, toxins, or autoimmune diseases can trigger myocarditis. In some cases, the etiology cannot be determined at all [35]. Myocarditis also occurs following other vaccinations, particularly following smallpox vaccination [4]. It is suspected that myocarditis caused by vaccines is the result of an autoimmune phenomenon. However, a verification of a causal relationship between myocarditis and COVID-19 vaccination is not yet possible.

Symptoms of myocarditis following other vaccinations have been reported after 1 week at the earliest. Due to the considerably earlier occurrence of myocarditis after COVID-19 vaccinations, the 1st vaccination, rather than the 2nd vaccination, is suspected to possibly trigger the autoimmune processes leading to myocarditis [45].

Regarding our investigated case of myocarditis, the period between the 2nd vaccination with a COVID-19 vaccine and the time of death was 11 h, which is also quite short. A causal relationship to vaccination was considered possible, but could not be proven beyond doubt.

The postmortem confirmation of myocarditis requires histologic examination of the heart and fulfillment of the Dallas criteria [5, 6]. Kytö et al. [36] reevaluated all cases of myocarditis diagnosed postmortem in Finland between 1970 and 1998. Among patients who died at a hospital, only one-third of the myocarditis cases had been diagnosed premortem. This result clearly emphasizes the importance of autopsies and histopathological examinations for the diagnosis of myocarditis not only for those who died at home but also for those who died at a hospital.

## Conclusions

The results of our study demonstrate the necessity of postmortem investigations on all fatalities following vaccination with COVID-19 vaccines. In 13 out of 18 cases, deaths were attributed to preexisting pathological changes. In these cases, extensive postmortem investigations did not reveal any evidence for known complications of vaccination with COVID-19 vaccines. In one case, myocarditis was determined to be the cause of death. A causal relationship to vaccination was considered possible, but could not be proven beyond doubt. In four cases, there was macromorphological evidence of VITT, which was confirmed by laboratory tests. Of those four, only one was diagnosed before death. Additional neuropathologic and histopathologic examinations revealed that VITT was the very likely cause of death in only two of the four cases. In order to identify a possible causal relationship between vaccination and death, in most cases an autopsy and histopathological examinations have to be combined with additional investigations, such as neuropathological examinations and laboratory diagnostics including platelet laboratory testings. This procedure requires close collaboration between several medical disciplines.
